# Investigation of Au/Si Eutectic Wafer Bonding for MEMS Accelerometers

**DOI:** 10.3390/mi8050158

**Published:** 2017-05-15

**Authors:** Dongling Li, Zhengguo Shang, Yin She, Zhiyu Wen

**Affiliations:** 1 National Key Laboratory of Fundamental Science of Novel Micro/Nano Device and System Technology, Chongqing University, Chongqing 400030, China; zhengry@cqu.edu.cn (Z.S.); Sheyin@cqu.edu.cn (Y.S.); wzy@cqu.edu.cn (Z.W.); 2 Key Laboratory of Optoelectronic Technologyand System of the Education Ministry of China, Chongqing University, Chongqing 400030, China; 3 Center for Inteligent Sensing Tchnology, College of Optoelectronic Engineering, Chongqing University, Chongqing 400030, China

**Keywords:** heating/cooling rate, contact force, adhesion layer, bonding strength, bonding interface, wafer-level packaging

## Abstract

Au/Si eutectic bonding is considered to BE a promising technology for creating 3D structures and hermetic packaging in micro-electro-mechanical system (MEMS) devices. However, it suffers from the problems of a non-uniform bonding interface and complex processes for the interconnection of metal wires. This paper presents a novel Au/Si eutectic wafer bonding structure and an implementation method for MEMS accelerometer packaging. The related processing parameters influencing the Au/Si eutectic bonding quality were widely investigated. It was found that a high temperature of 400 °C with a low heating/cooling rate of 5 °C/min is crucial for successful Au/Si eutectic bonding. High contact force is beneficial for bonding uniformity, but the bonding strength and bonding yield decrease when the contact force increases from 3000 to 5000 N due to the metal squeezing out of the interface. The application of TiW as an adhesion layer on a glass substrate, compared with a commonly used Cr or Ti layer, significantly improves the bonding quality. The bonding strength is higher than 50 MPa, and the bonding yield is above 90% for the presented Au/Si eutectic bonding. Furthermore, the wafer-level vacuum packaging of the MEMS accelerometer was achieved based on Au/Si eutectic bonding and anodic bonding with one process. Testing results show a nonlinearity of 0.91% and a sensitivity of 1.06 V/g for the MEMS accelerometer. This Au/Si eutectic bonding process can be applied to the development of reliable, low-temperature, low-cost fabrication and hermetic packaging for MEMS devices.

## 1. Introduction

Wafer bonding is as a key technology for microelectronics and micro-electro-mechanical systems (MEMS) for creating three-dimensional (3D) structures and hermetic packaging. Several bonding techniques have been developed in the last several decades, including silicon fusion bonding, anodic bonding, solder bonding, adhesive bonding, and eutectic bonding [1,2,3]. Compared with other bonding technologies, Au/Si eutectic bonding benefits from a low eutectic temperature (363 °C), being non-sensitive to the roughness of bonding surface and particles, having good mechanical stability, and compatibility with aluminum interconnect [4]. Therefore, it is considered the most important approach to establishing strong bonds and hermetic seals, and is widely used in the fabrication of MEMS devices, 3D interconnects, and wafer-level vacuum packaging [5,6]. As such, Au/Si eutectic bonding has been investigated extensively. Maryam Abouie et al. [7] found craters on the bonding interface and attributed them to the anisotropic and non-uniform reaction between Au and Si. Michael David Henry and Catalina R. Ahlers [8] studied the effect of platinum diffusion barriers and the metallization process in Au/Si eutectic bonding. They concluded that the barrier broke down at approximately 375 °C and resulted in uncontrolled stoichiometry variations and the creation of micro voids. Therefore, the bond strength and hermeticity were reduced, but the alternative material was not presented. Errong Jing et al. [9] investigated the interface of Au/Si (100) eutectic bonding with an infrared (IR) microscope and related their observations to the bond strength. The results showed that a strong bond had many square black spots in the IR images, whereas a poor bond had fewer or no square black spots. This method provided a convenient method using an IR microscope to evaluate bonding quality without destroying the bonded pairs. Furthermore, the method for improving the reliability of Au/Si eutectic bonding with either an Mo-buffered layer [10] or localized bonding [11] was also studied. However, there have been few reports about the influence of contact force and the heating/cooling rate on Au/Si eutectic bonding quality. In addition, new stacks of metal layers are still needed for simplifying the fabrication process and enhancing the bonding quality. 

In this work, we developed a simple Au/Si eutectic bonding process that enabled high bonding strength and a uniform bonding interface for hermetic packaging. The influences of process parameters (such as bonding temperature, contact force, and heating/cooling rate) on the bonding strength and bonding interface were studied. Pyrex 7740 glasses with patterned Cr/Au, Ti/Pt/Au, or TiW/Au layers were bonded to Si wafers. The bonding interfaces were observed and analyzed with an optical microscope, a scanning electron microscope (SEM), and a related energy dispersive spectrometer (EDS). The bonding strength and bonding yield were evaluated with a tensile pulling test and a dicing experiment, respectively. Finally, a wafer-level vacuum packaging structure was designed for an MEMS accelerometer, which achieved hermetic packaging and a metal wire interconnection with a single bonding process, and the performances of the accelerometer were examined. 

## 2. Au/Si Eutectic Bonding

Au/Si eutectic bonding is a kind of intermediate layer wafer bonding that utilizes the low melting temperature of an Au/Si eutectic alloy. The principle of Au/Si eutectic bonding is quite similar to soldering. A liquid phase is formed when the temperature exceeds the eutectic point, and Au and Si are then mixed together with a certain ratio. Subsequently, the material mixtures are solidified regardless of whether the temperature decreases below the eutectic point or whether the ratio of the concentration leaves the liquid area. The reaction starts with atomic contact of the partners; with an increase in temperature, the diffusion of Au and Si occurs. As soon as the eutectic mixture reaches the eutectic composition, the liquid phase begins, and further mixing and diffusion processes are accelerated. From the phase diagram of Au/Si [12], it can be seen that the melting point of the Au/Si eutectic alloy is around 363 °C, which is much lower than that of the melting points of Au (1063 °C) and Si (1412 °C) respectively. The eutectic composition is 19 atom % (3 wt %) Si and 81 atom % (97 wt %) Au.

The layer thicknesses of Au and Si which are involved in the eutectic reaction can be calculated as follows:

(1) $$\frac{d_{\mathrm{Au}}}{d_{\mathrm{Si}}}=\frac{m_{\mathrm{Au}}/\rho_{\mathrm{Au}}}{m_{\mathrm{Si}}/\rho_{\mathrm{Si}}}=\frac{m_{\mathrm{Au}}\rho_{\mathrm{Si}}}{m_{\mathrm{Si}}\rho_{\mathrm{Au}}}=\frac{3.9}{1}$$

where *d*_Au_ and *d*_Si_ are the thickness of Au and Si layers, respectively. *m*_Au_ and *m*_Si_ are the relative weight percentages of Au and Si at the eutectic point, respectively. *ρ*_Au_ and *ρ*_Si_ are the density of Au and Si, respectively.

Otherwise, the thickness of Au/Si eutectic alloy is associated with the bonding temperature and bonding time. The empirical equation is given by [13]

(2) $$h\approx c(T-T_{c})t^{1/2}$$

where *h* is the thickness of Au/Si eutectic alloy (μm), *T* is the bonding temperature (K), and *T_c_* is the eutectic temperature (K). *t* is the bonding time (h), *c* is a constant related to the bonding material, with a value of 0.065 μm/(h^1/2^·K) for Au–Si eutectic formation. It is obvious that the bonding strength becomes higher and the final thickness of Au/Si alloy becomes thicker when the bonding temperature and bonding time increase. 

## 3. Experimental Details

### 3.1. Wafer Preparation

**Figure 1:**
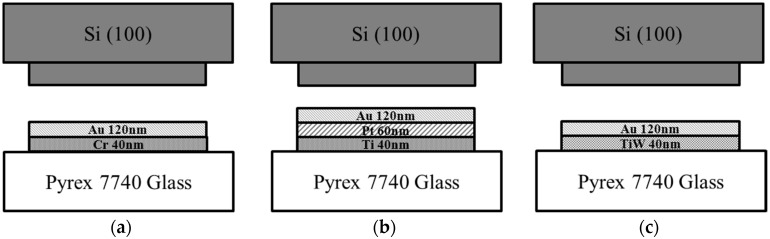
Schematic diagram of Au/Si eutectic bonding structures: Si structures bonded with patterned (**a**) Cr/Au, (**b**) Ti/Pt/Au, and (**c**) TiW/Au on Pyrex 7740 glass wafer.

Four inch double-polished n-type (100) Si wafers with resistivity around 2–4 Ω·cm (Dingjing Co., Ltd., Luoyang, China) and Pyrex 7740 glass wafers (Taikunisi Co., Ltd., Suzhou, China) were used for bonding experiments. Figure 1 illustrates the Au/Si bonding structures studied in this work. The Pyrex 7740 glass was immersed in H_2_SO_4_:H_2_O_2_ (3:1) at 120 °C for 10 min to remove organic particles. The bonding layer of Cr/Au, Ti/Pt/Au, or TiW/Au were deposited on the glass substrate in sputtering system FHR-MS100 × 6 (FHR Anlagenbau Gmbh, Dresden, Germany) with a base pressure of 1 × 10^−6^ mbar. The bonding structures on the Si wafer were fabricated by a standard wet etching process. 

### 3.2. Bonding Process

Before bonding, the Si wafer with a bonding structure and the Pyrex 7740 glass wafer with a deposited metal layer were wet-cleaned with fuming nitric acid (a kind of concentrated nitric acid with the nitric acid content of 90–98%) at 100 °C for 5 min to remove organic contaminations. The wafers were then rinsed in DI water and dried with nitrogen. In addition, the Si wafer was dipped in 1:100 HF for 10 min in order to remove the native oxide layer on the Si surface and ensure small surface roughness simultaneously. Immediately after DI water rinsing and nitrogen drying, the wafer pairs were aligned by a mask aligner MA6/BA6 (Karl Suss, Munich, Germany) and then loaded into a Karl Suss vacuum bonder SB6e (Karl Suss, Munich, Germany). After the N_2_ purge, the bonder was pumped to a base vacuum of 5 × 10^−5^ mbar, and then ramped to the bonding temperature. The eutectic bonding was carried out at 380–400 °C with an applied force of 2000–5000 N, and then slowly cooled down to room temperature.

### 3.3. Bonding Quality Evaluation

The bonded wafer was cut into 7.14 mm× 7.14 mm pieces at a speed of 1 mm/s with the dicing saw DS616. The valid bonding areas are 5.49 × 10^−6^ m^2^ of a single chip and 8 × 10^−4^ m^2^ on the entire wafer. The dicing results can be used to initially evaluate the bonding quality: the separated pieces are considered to be a failed bonding, and the pieces staying together represent successful bonding. Bonding yield is defined as the percentage of successfully bonded pieces on each wafer. After the dicing test, surviving bonded pieces went with a tensile pulling test to determine bonding strength. A force was continuously and perpendicularly applied to the bonding interface until the sample was broken. The bonding strength was determined by dividing the maximum force to the bond area. A similar behavior was detected by razor blade tests for preliminarily estimating bonding strength. A thin razor blade was inserted between the bonded pairs from the wafer edges, and the separated surfaces were inspected with an optical microscope and analyzed with an energy dispersive spectrometer (EDS, ZEISS, Jena, Germany). Either the silicon broke due to a high bonding strength or delamination at the Au/Si interface occurred due to a weak bonding strength. The fracture surfaces were also examined by a scanning electron microscope EV018 (SEM, ZEISS, Jena, Germany).

## 4. Results and Discussion

### 4.1. Temperature

Au/Si eutectic bonding is an inter-diffusion process between Au and Si, and heat plays a major role in this process. When bonding temperature exceeds the eutectic point (363 °C), Au and Si are in contact with each other, and the liquid phase alloy with eutectic composition can be formed during the diffusion process. As time goes on, the liquid phase layer becomes thick. During the subsequent cooling process, the liquid phase continuously alternates with these two kinds of metals, each of which is grown, crystallized, and precipitated on the basis of its original solid phase, respectively. Thus, the eutectic alloy between the two materials binds the two wafers together tightly.

**Figure 2:**
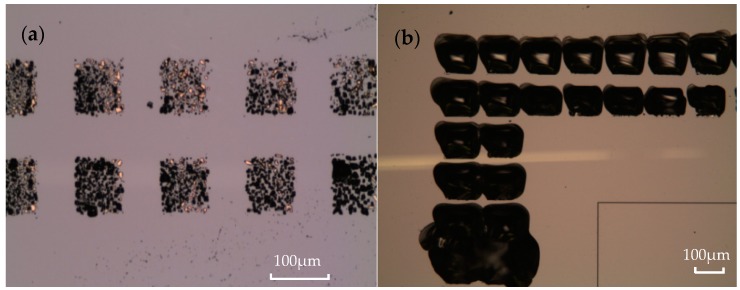
Optical microscope pictures of separated glass surface at different bonding temperature. (**a**) Bonding at 380 °C, some Si particles could be seen; (**b**) bonding at 400 °C, big pieces of silicon peeled off from bulk Si.

The influence of bonding temperature on Au/Si eutectic bonding is examined and analyzed. With the bonding temperature of 380 °C, the bonding strength is 6.7 MPa. It is so weak that the bonded wafers are easily separated during the blade test. Observing the separated surfaces, a slight in-depth diffusion of silicon into gold already occurred, and some silicon particles are seen on the separated surface, as shown in Figure 2a. However, the bonding quality is insufficient for the further applications, especially for vacuum packaging. Thus, the bonding temperature has been raised to 400 °C, and the measured bonding strength is 66.8 MPa. Big pieces of silicon are peeled off from bulk silicon after the blade test, which indicates that the bonding strength is higher than the bulk Si, as shown in Figure 2b. It can be concluded that a high bonding temperature of 400 °C is needed for high bonding strength. According to S. Lani et al. [14], the high bonding temperature is mainly needed due to the impurity elements in the bonding materials (such as oxygen and hydrogen), or the temperature non-uniformity on the whole wafer, which causes a partial reaction between Au and Si. In addition, the difference between the setting temperature of SB6e and the actual temperature is another factor for a higher bonding temperature. As expected, the bonding strength increases with a further increase in bonding temperature, which is shown in Table 1 (Samples 3 and 8). From Equation (2), a higher bonding temperature results in thicker Au/Si alloys, so the bonding strength increases. However, a high bonding temperature always causes metal wire melting or performance declining for temperature-sensitive devices [15]. Therefore, the optimized bonding temperature is 400 °C. 

### 4.2. Heating/Cooling Rate

**Figure 3:**
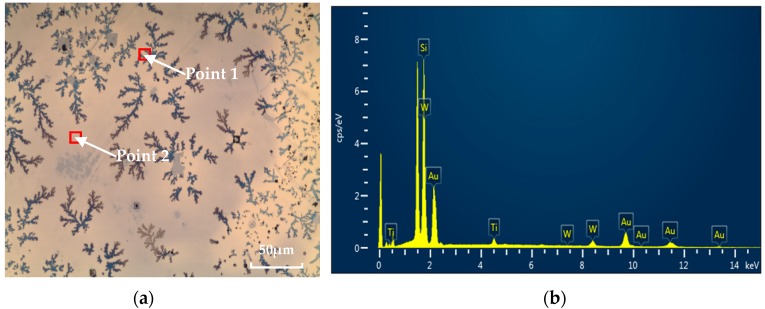
Failed bonding with high heating/cooling rate. (**a**) Separated surface of glass substrates after the blade test; (**b**) component analysis at Point 1.

The influence of temperature heating/cooling rate on successful eutectic bonding was also investigated. Figure 3a exhibits a failed bonding at high heating/cooling rate of 15 °C/min. The bonded wafers were easily separated by the blade test from the bonded interface. Similar to the observation by Bokhonov et al. [16], some dendrite crystallizations were seen on the separated Au surface. The component elements on the surface were analyzed via EDS. Results show that the chemical elements were as follows: Si (29.82 wt %), Au (52.72 wt %), Ti (2.8 wt %), and W (14.67 wt %) at Point 1, as shown in Figure 3b, whereas the chemical elements of Point 2 are as follows: Si (10.87 wt %), Au (69.46 wt %), Ti (3.43 wt %), and W (16.24 wt %). Both analyses ignored the influence of carbon (C) and oxygen (O) elements. A small amount of silicon at Point 2 was introduced by the glass substrate. The increase in Si content at Point 1 indicates that a liquid Au/Si alloy was formed. However, the bonding strength is weak because of the formation of dendrites. Au/Si eutectic bonding is a process of liquid phase transformation into a solid phase, and the crystallization occurred during the solidification process. With a high heating/cooling rate, the atoms in the liquid phase can diffuse easily, whereas the atoms in the solid phase are strained to spread due to a lower diffusion rate. Thus, the composition of the later crystal is different from that of the previous crystal, resulting in the formation of dendrites. The dendrites will cause the crystallographic direction to deviate from its previous growing direction and will hinder further solidification of the Au/Si alloy. As a result, a stable and continuous Au/Si alloy cannot be formed. On the other hand, a low heating/cooling rate prolongs the solidification process, indicating a longer liquid alloy time. Therefore, the liquid Au/Si alloy is easily spreads to a non-bonded region (called metal squeezing), especially at high contact force, which will be discussed in the next section. Furthermore, the electrical connection of devices can be damaged because of a serious metal squeezing. 

**Figure 4:**
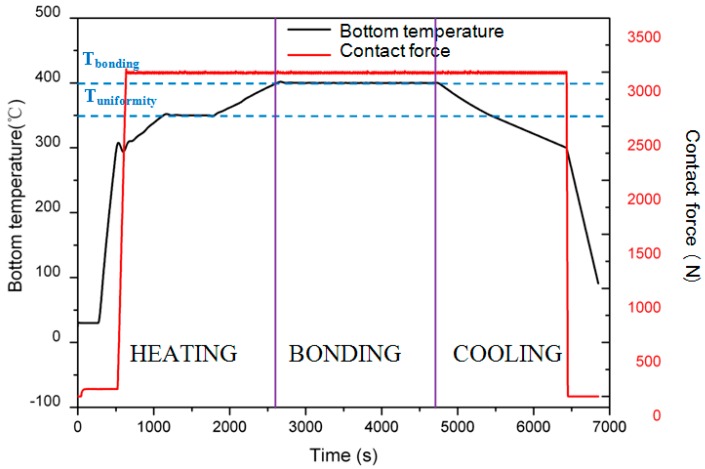
The optimized temperature curve of Au/Si eutectic wafer bonding.

The optimized temperature curve of Au/Si eutectic bonding is shown in Figure 4. First, the samples are heated up from room temperature to 300 °C with the maximum power of SB6e. Then, the temperature rises to 350 °C and remains constant for 10 min for uniform heating of both substrates. Subsequently, the samples are heated to 400 °C with a heating rate of 5 °C/min, which is maintained for 20 min. Finally, the cooling rate is controlled to 5 °C/min for the full solidification of Au and Si. The contact force is applied as the temperature above 300 °C, and the samples are heated from both the top and the bottom at the same time. 

### 4.3. Contact Force

**Figure 5:**
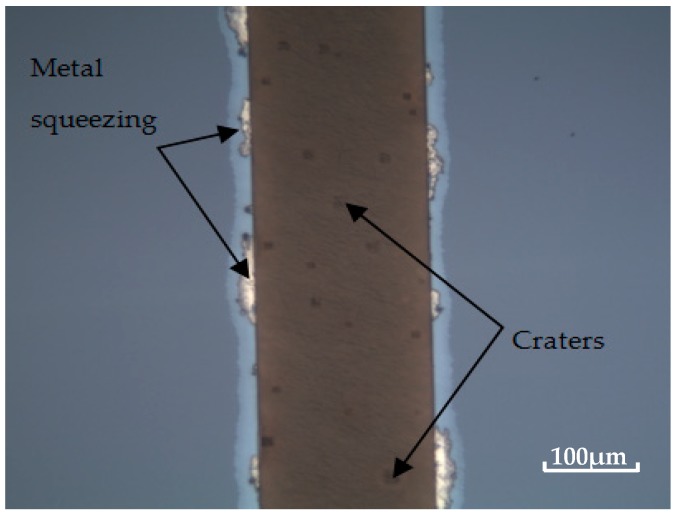
Metal squeezing and craters appear at the bonding interface with a high contact force of 5000 N.

Meanwhile, the influence of the contact force on bonding quality is also examined, as shown in Table 1 (Samples 2, 3, and 6). It can be seen that the bonding strength increases from 37.5 to 66.8 MPa, as the contact force varies from 2000 to 3000 N. On the one hand, high contact force can overcome the non-planar and rough surface, ensuring good contact of Au and Si surfaces. On the other hand, a high contact force is beneficial for the disruption of a native oxide layer at some spots [17]. Thereby, more exposing local sites in the underlying Si diffuse into the Au. The Si on the direct contact region will dissolve into the Au and diffuse rapidly along the grain boundaries of the Au film, so the bonding strength will increase. However, with the further increase in contact force to 5000 N, the bonding strength decreases to 41.4 MPa. This is because a high contact force results in a metal squeezing out of the interface, causing a poor interface layer uniformity, and craters appear at the interface, as shown in Figure 5. Nevertheless, the bonding interface is uniform and no metal is squeezed with a 3000 N contact force. 

Furthermore, observing the deviation of the tensile strength in Table 1, it can be concluded that high contact force increases the uniformity of the Au/Si reaction and improves the bonding yield, which is also attributed to the tight contact of the Au and Si surfaces. In addition, the bonding strength slightly increases with the increase in bonding time, from 66.8 to 74.3 MPa, with the bonding time increasing from 20 to 40 min, as shown in Table 1 (Samples 3 and 7). However, a high bonding time also increases the risk of reflow of the Au/Si alloy [18]. Therefore, the bonding time must be adjusted according to specific applications. In summary, the optimal contact force for the designed structure is 3000 N, and the bonding time is 20 min. 

### 4.4. Adhesion Layer

**Figure 6:**
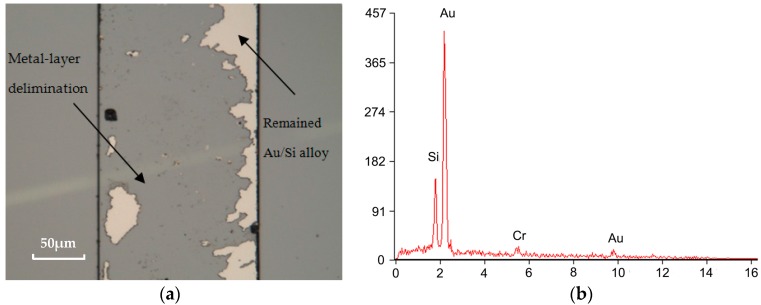
Optical photograph and component analysis of separated surface for Cr/Au layer. (**a**) Metal layer peeling off of the substrates; (**b**) component analysis of Au/Si alloy.

Metallization layers such as Cr and Ti are typically deposited on the substrate to ensure good adhesion, and Pt has been used in conjunction with the adhesion layers as a diffusion barrier between Au and the substrates [19]. However, during Au/Si eutectic bonding at 400 °C, these materials tend to diffuse into Au or Au/Si alloy. Therefore, there are no adhesion layers left and the adhesive function disappears, resulting in the delamination of Au or Au/Si alloy from the substrates, as shown in Figure 6. Analyzing the alloy composition of the Cr/Au structure, it contains 5.07 wt % Si, 91.9 wt % Au and 3.03 wt % Cr, which is different from the ideal Au/Si alloy. This can be attributed to the formation of a silicon component (CrSi_2_) caused by the interaction between Cr and Si [20]. 

**Figure 7:**
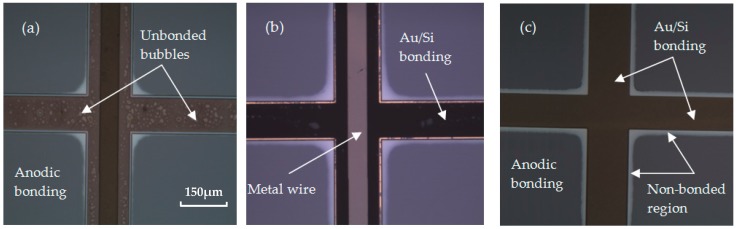
Optical microscope images for different metal layers. (**a**) Bonding with a Cr/Au layer, unbonded bubbles appeared in the bonding interfaces; (**b**) bonding with a Ti/Pt/Au layer, color of the metal layer changed due to the breakdown of the Pt barrier; (**c**) bonding with a TiW/Au layer, a stable bonding interface is achieved.

TiW is a kind of composite refractory metal with the composition of Ti 10 wt % and W 90 wt %, and may act as an alternative material for successful Au/Si eutectic bonding. TiW is not only a good adhesion layer, but also a diffusion barrier preventing inter-diffusion between metals [7]. The bonding performances with a different adhesive layer are estimated by an optical microscope inspecting from the backside of Pyrex 7740 glass, as shown in Figure 7. It can be seen that, for the Cr/Au layer, many unbonded bubbles appear in the bonding interfaces, resulting in a non-uniform bonding interface. The formation of voids can be attributed to the delimitation of the metal layer from glass substrates, caused by the diffusion of Si into a Cr layer, as analyzed above. Although the unbonded bubbles are greatly reduced by using a Ti/Pt/Au layer, the color of the metal layer changed substantially due to the breakdown of the Pt barrier at high temperature. Thus, diffusion of Si into the Ti layer occurs, and the bond strength and hermeticity are reduced. Instead, with the TiW layer, the bonding interfaces are stable, and neither significant voids nor any inter-diffusion of metal is observed. 

**Figure 8:**
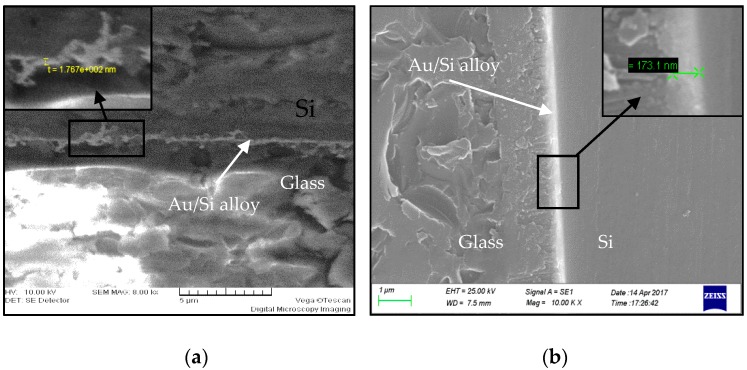
Cross-sectional SEM images of Au/Si bonding interfaces. (**a**) Bonding with a Ti/Pt/Au (40 nm/60 nm/120 nm) layer, and the front of bonding interface is irregular; (**b**) bonding with a TiW/Au (40 nm/120 nm) layer, and the front of bonding interface is straight and continuous.

Figure 8 illustrates the cross-sectional SEM images of the Au/Si bonding interfaces with Ti/Pt/Au and TiW/Au layers. Bright bonding interfaces are visible in both samples, indicating the formation of an Au/Si alloy. However, the front of the bonding interface is irregular with a thickness of 176.7 nm for the Ti/Pt/Au layer. The nonuniformity of the bonding interface is mainly due to the inter-diffusion between metal layers and the substrates. Otherwise, a high contact force also causes metal squeezing, and results in a decrease in Au/Si alloy thickness. However, the front of the bonding interface of TiW/Au is straight and continuous, exhibiting a high bonding strength of 66.8 MPa. Compared to the TiW/Au thickness of 40 nm and 160 nm, respectively, the thickness of the Au/Si alloy is slightly increased due to the diffusion of Si into Au layers. 

Finally, the repeatability of Au/Si eutectic bonding with the TiW/Au layer is investigated with a bonding temperature of 400 °C, an applied force of 3000 N, a heating/cooling rate of 5 °C/min, and a bonding time of 20 min. All samples exhibit a uniform bonding interface. The bonding strength varies from 57.6 to 67.8 MPa, as shown in Table 1 (Samples 3, 4, and 5). The results show good repeatability of the Au/Si eutectic bonding process, which are beneficial for further applications. 

## 5. The Application of Au/Si Eutectic Bonding in an MEMS Accelerometer

### 5.1. Package Design

**Figure 9:**
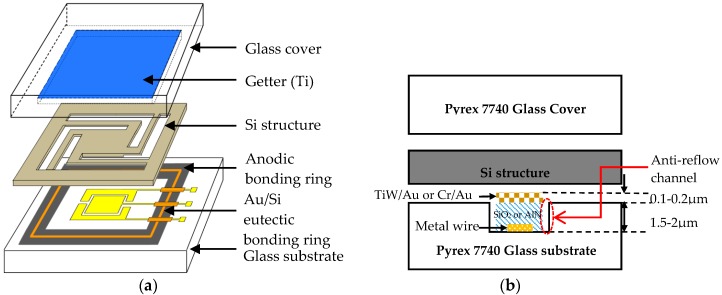
Schematic diagram of wafer-level vacuum packaging for an MEMS accelerometer. (**a**) 3D structure of packaged MEMS accelerometer; (**b**) a cross-sectional diagram of accelerometer observing from the metal wire section.

Figure 9a shows the three-dimensional (3D) structure of the wafer level packaging for the MEMS accelerometer. It is a typical sandwich structure of glass–Si–glass. The middle Si device wafer forms the accelerometer structure as well as the sealing wall, and it is bonded to the glass substrate by anodic bonding and Au/Si eutectic bonding. The metal electrode and metal wire are deposited on the glass substrate for voltage supply and signal transmission, and the bonding rings for hermetic packaging and metal wire interconnection. The glass cover with a patterned groove and Ti getter is bonded with an Si device wafer via anodic bonding, which provides a vacuum chamber and ensures long-term stability of vacuum. Figure 9b illustrates the cross-section diagram of the MEMS accelerometer observing from the metal wire section. The metal wire and the Au/Si eutectic bonding ring are separated by an insulation layer of SiO_2_ or AlN with a thickness of more than 1.5 μm. The anti-reflow channels are arranged at the edge of the Au/Si bonding ring, preventing the reflow of metal squeezing to the device area. Otherwise, the Au/Si eutectic bonding ring is higher than that of an anodic bonding ring of 0.1–0.2 μm, which ensures good contact of bonding rings with the Si device layer and minimizes the non-bonded region on the edge of Au/Si eutectic bonding ring (as shown in Figure 7c). This structure can achieve hermetic packaging and metal wire interconnection in a single bonding process due to the similar processing temperature of Au/Si eutectic bonding and anodic bonding.

### 5.2. Fabrication Process

**Figure 10:**
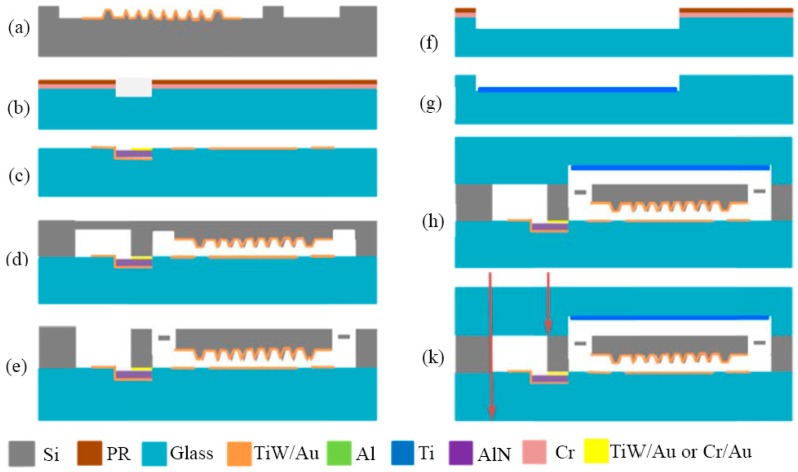
Major fabrication process flow for the MEMS accelerometer.

The main steps for the fabrication of the MEMS accelerometer are presented in Figure 10. The process starts with the wet etching of the structure on an Si substrate, and a 40 nm/120 nm TiW/Au layer is then sputtered to form the sensitive structure and cathode (Figure 10a). A 1.5-μm-thick groove is etched on the Pyrex 7740 glass using Cr and photoresist (PR) as the etching mask (Figure 10b). The electrodes and metal wire are fabricated by a lift-off process, and a 1.5-μm-thick SiO_2_ or AlN is deposited between the metal wire and the Au/Si eutectic bonding ring for isolation purposes (Figure 10c). The Si device layer and the Pyrex 7740 glass are bonded together by anodic bonding and Au/Si eutectic bonding at 400 °C, with an applied voltage of −1000 V (Figure 10d). The thickness of the Si device layer is determined by thinning the Si wafer to 85 μm, and the devices are then released via deep reactive ion etching (Figure 10e). A 10-μm-deep cavity on the glass cover is also etched with a buffered HF solution (Figure 10f), and a 500 nm Ti getter is deposited inside the cavity by sputtering (Figure 10g). The Ti getter is inherently activated during the wafer level packaging process with anodic bonding at 350 °C (Figure 10h). Finally, the acceleration chips are separated by a dicing process with dimensions of 6.1 mm × 7.5 mm (Figure 10k).

**Figure 11:**
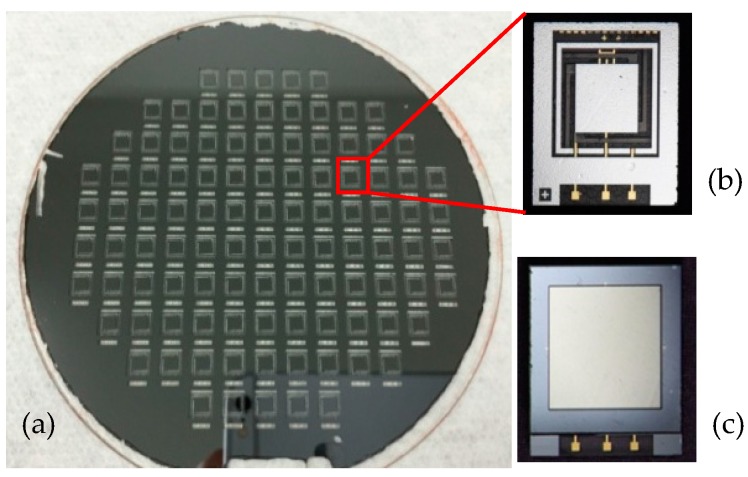
Photographs of fabricated accelerometers. (**a**) The chips on the whole wafer; (**b**) a signal chip without glass cover; (**c**) the hermetically packaged chip.

Figure 11 shows the photographs of fabricated accelerometers with and without glass cover. The bonded wafer exhibits high bonding strength that can survive the following thinning and dicing processes with no pieces peeling off. 

### 5.3. Performance of the Accelerometer

**Figure 12:**
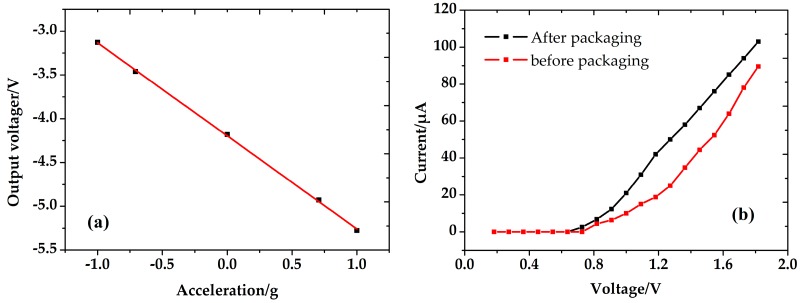
Performance of the accelerometer. (**a**) The output fitting curve of acceleration and output voltage; (**b**) *I*/*V* characteristic of accelerometer.

The performances of the packaged accelerometer are shown in Figure 12. The nonlinearity of the accelerometer is 0.91% with an acceleration from −1*g* to +1*g*, and the sensitivity is 1.06 V/g. Furthermore, the *I*/*V* characteristic of the accelerometer both before and after packaging are analyzed. It is shown that the emission current is increased from 52.4 to 76 μA after vacuum packaging, which is mainly due to the improvement of the vacuum. The results indicate that the designed structure for vacuum packaging is feasible. However, efforts on the long-term preservation and reliability of vacuum are still needed in further work.

## 6. Conclusions

In this paper, the Au/Si eutectic bonding method and its application in MEMS accelerometer packaging are investigated. For a high bonding strength and a uniform bonding interface, the processing parameters of Au/Si eutectic bonding were studied and optimized. The bonding temperature and heating/cooling rate were considered to be the key parameters influencing the Au/Si eutectic bonding quality. The bonding temperature needs to be set to 400 °C with a heating/cooling rate of 5 °C/min for stable Au/Si eutectic bonding. High contact force is beneficial to bonding uniformity, but the bonding strength and bonding yield decrease when the contact force increases from 3000 to 5000 N due to the metal squeezing out of the interface. Bonding of the TiW/Au layer exhibited a much better performance than did the Cr/Au or Ti/Pt/Au layers, and the bonding strength was higher than 50 MPa, with a bonding yield above 90%. Particularly, the wafer-level vacuum packaging for the MEMS accelerometers was achieved based on Au/Si eutectic bonding and anodic bonding, which can achieve hermetic packaging and metal wire interconnection simultaneously. The testing results show a nonlinearity of 0.91% and a sensitivity of 1.06 V/g for the MEMS accelerometer.

## Figures and Tables

**Table 1:** Bonding quality of different parameters.

No.	Temperature (°C)	Contact Force (N)	Pressure (MPa)	Time (min)	Bonding Strength (MPa) ^1^	Bonding Yield (%)
1	380	3000	3.5	20	6.7 ± 1.2 (18%)	23
2	400	2000	2.3	20	37.5 ± 3.8 (11%)	67
3	400	3000	3.5	20	66.8 ± 4.6 (7%)	91
4	400	3000	3.5	20	57.6 ± 5.6 (9%)	92
5	400	3000	3.5	20	62.4 ± 6.2 (10%)	90
6	400	5000	5.8	20	41.4 ± 2.1 (5%)	85
7	400	3000	3.5	40	74.3 ± 4.5 (6%)	93
8	420	3000	3.5	20	84 ± 6.8 (8%)	88

^1^ An average tensile strength and standard deviation of five samples of one wafer pair.
